# Graphene-Based Sensors for Food Freshness Monitoring: Recent Advances, Performance, and Practical Challenges

**DOI:** 10.3390/s26165278

**Published:** 2026-08-20

**Authors:** Grazia Giuseppina Politano

**Affiliations:** Department of Environmental Engineering, University of Calabria, 87036 Rende, Italy; grazia.politano@unical.it

**Keywords:** graphene, food packaging, food freshness

## Abstract

Food spoilage along the supply chain represents a major global challenge, contributing to economic losses, environmental impacts, and food safety concerns. Graphene-based materials have emerged as promising platforms for real-time food freshness monitoring owing to their high surface area, electrical conductivity, chemical sensitivity, and compatibility with flexible sensing architectures. This review critically examines graphene-based sensing strategies for food freshness and spoilage monitoring, including chemiresistive, dielectric, field-effect, optical/fluorescence, photoelectrochemical, mass-sensitive, and colorimetric approaches. Representative sensing platforms are compared in terms of analytical performance, including detection range, limit of detection, selectivity, response and recovery times, calibration, reproducibility, and stability. Particular attention is given to machine-learning-assisted sensing, multifunctional platforms for temperature, humidity, and gas monitoring, and the challenges associated with real-world implementation, including environmental interference, sensor fouling, signal drift, long-term stability, and cross-matrix validation. Finally, commercialization readiness, integration into intelligent packaging, and safety and regulatory considerations related to graphene-based food-contact applications are discussed. Overall, the review identifies the main technological gaps and future research priorities toward robust, scalable, and practical graphene-based systems for real-time food freshness monitoring.

## 1. Introduction

Food spoilage along the supply chain represents a major global challenge, contributing significantly to economic losses, environmental impact, and resource inefficiency [1]. The need for safer, longer-lasting, and more sustainable food preservation technologies has therefore driven increasing interest in innovative approaches for real-time monitoring of food quality [2]. In this context, the development of intelligent packaging systems capable of providing non-destructive, in situ, and continuous assessment of freshness has emerged as a key research direction [3].

Graphene [4], a single-atom-thick carbon sheet arranged in a two-dimensional hexagonal lattice, exhibits an exceptional combination of physical, mechanical, and functional properties. Moreover, graphene is highly transparent (~97.7% optical transmittance) [5] and lightweight, allowing its integration into flexible and transparent packaging systems without compromising visual inspection of the product [6].

Graphene-based materials have attracted particular attention in this field due to their high surface area, excellent electrical conductivity, and chemical sensitivity that make them particularly suitable for detecting changes associated with food spoilage [7].

**Figure 1:**
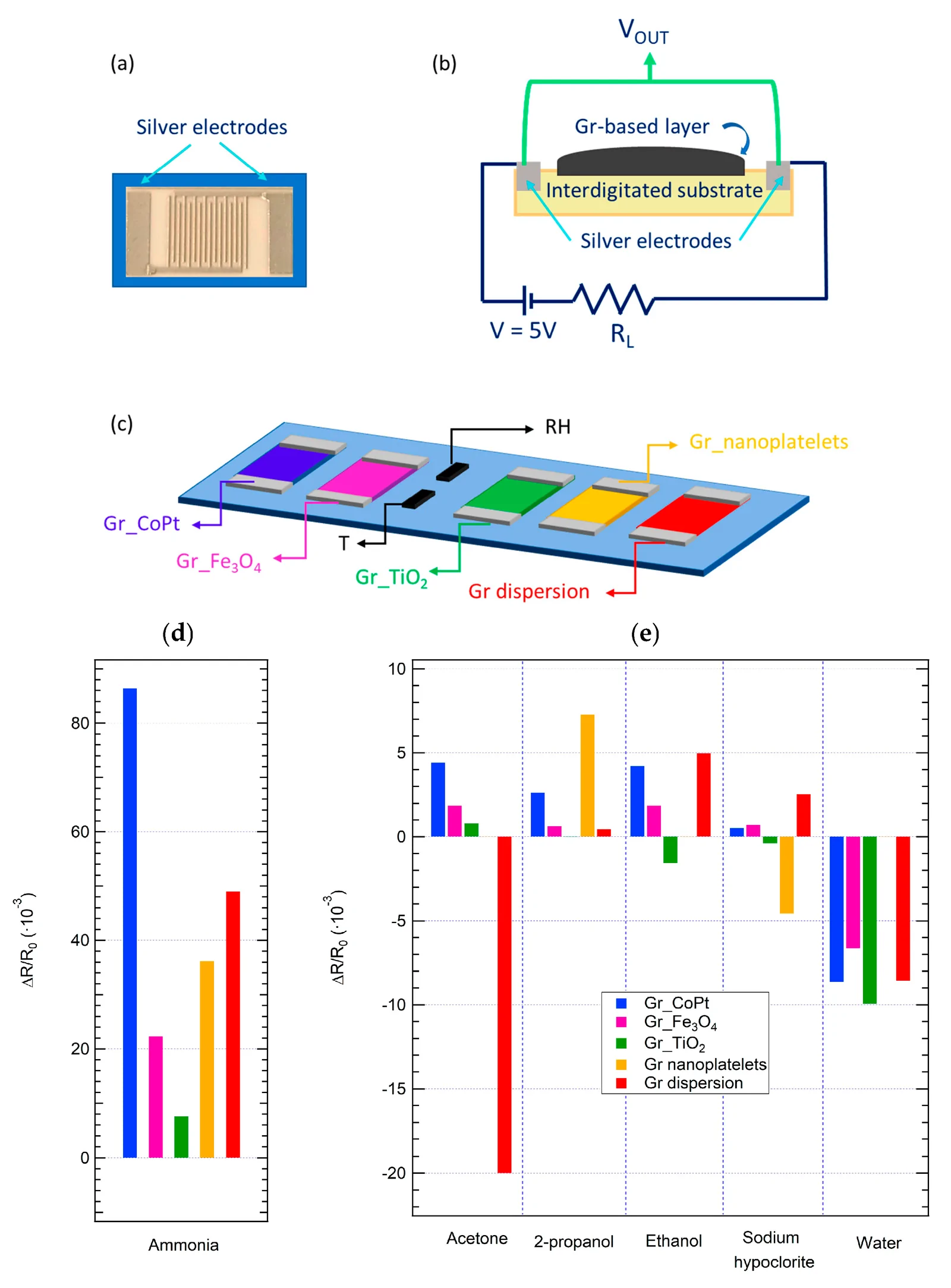
Chemiresistive graphene-based sensor array. (**a**) Bare interdigitated alumina substrate with silver electrodes. (**b**) Electrical readout scheme for the individual chemiresistor sensors. (**c**) Sensor-array layout including graphene (Gr) dispersion, graphene nanoplatelets, Gr–TiO_2_, Gr–Fe_3_O_4_, and Gr–CoPt sensing elements, with commercial relative humidity (RH) and temperature sensors integrated in the central region. Sensor-array response profiles recorded upon exposure to selected target vapors. (**d**) Response to ammonia. (**e**) Responses to acetone, 2-propanol, ethanol, sodium hypochlorite, and water. Adapted from Ref. [8].

**Figure 2:**
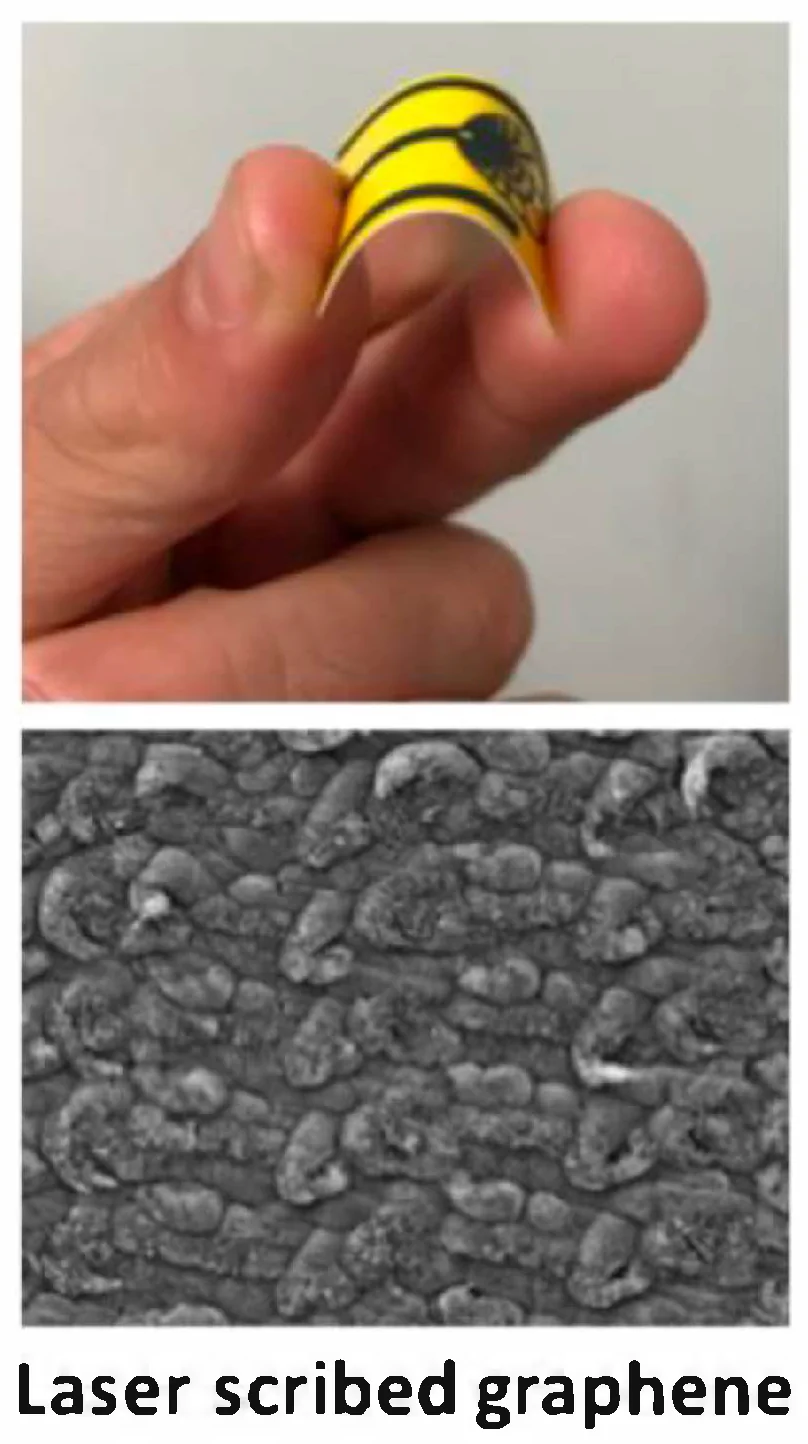
Schematic representation of a low-cost, disposable graphene-based nanobiosensor designed for the detection of biogenic amines in food. The device was fabricated using locally available materials and its performance was benchmarked against sensors prepared with analytical-grade components. Adapted from Ref. [9].

As a result, graphene and its derivatives have been widely explored for sensing applications including volatile organic compounds (VOCs) [8], ammonia [8] (see Figure 1) and biogenic amines [9] (see Figure 2), which are commonly associated with degradation processes in food systems.

The current literature reflects a wide diversity of sensing approaches. Several studies focus on colorimetric systems, enabling direct visual detection of spoilage through pH-dependent color changes [10]. Other works investigate electronic sensing platforms [11], which provide quantitative detection of VOCs through changes in electrical properties. In addition, alternative strategies such as fluorescence-based sensing [12] and electronic nose systems [13] have been proposed, highlighting the versatility of graphene-based materials in different sensing configurations.

Recent literature on graphene-based functional nanomaterials further clarifies why nanoscale engineering is central to sensing performance. Graphene quantum dots (GQDs) [14], as zero-dimensional graphene derivatives, exhibit optical and electronic properties that can be tuned through size control, quantum confinement, edge structure, defects, heteroatom doping, and surface functionalization. These structural parameters affect bandgap, photoluminescence, charge-transfer processes, and analyte–surface interactions, which are key factors in determining sensor sensitivity and selectivity. In GQD-based fluorescent sensors, target molecules may modulate the signal through mechanisms such as photoinduced electron transfer, energy transfer, inner-filter effects, aggregation-induced quenching, or fluorescence recovery after specific analyte binding. In the context of food-quality assessment, graphene-assisted optical sensing has already been demonstrated using graphene-enhanced Raman spectroscopy (GERS) [15]. For example, Gopal et al. [16] demonstrated the use of gold- and silver-nanoparticle-mediated GERS for evaluating the freshness of fruits and vegetables. In this approach, graphene/metal-nanoparticle platforms enhanced Raman signals from food surfaces and enabled the distinction between market-fresh and refrigerated samples through storage-dependent spectral changes. The sensing response was attributed to the combined contribution of graphene and noble-metal nanoparticles, including fluorescence-background suppression by graphene and plasmonic enhancement by Au or Ag nanoparticles.

Beyond the sensing mechanism, the choice of the functional material also strongly influences sensor performance and practical applicability. In this respect, graphene-based materials can be compared with other emerging platforms such as MXenes [17] and MOFs [18]. Graphene offers high electrical conductivity and mechanical flexibility, which facilitate rapid electrical transduction and integration into flexible sensing devices [19], whereas MOFs offer high porosity and selective molecular adsorption [20]. However, MOFs may suffer from poor intrinsic electrical conductivity and structural instability, while hybridization with graphene can help overcome these limitations [21]. Graphene-derived nanomaterials can improve the structural stability and electrical conductivity of MOFs while limiting graphene restacking and aggregation, thereby broadening the potential applications of MOF–graphene hybrids in food-contaminant sensing [22]. Thus, the main advantage of graphene lies not only in its intrinsic electrical and mechanical properties, but also in its versatility as a conductive platform for the development of hybrid sensing architectures.

In this context, the present review focuses on graphene-based sensors for food freshness and spoilage monitoring, with particular attention to the relationship between sensing mechanisms, material properties, and practical implementation.

Although several recent reviews have addressed graphene-based sensors for food applications, most have focused on specific aspects, such as microbial detection, electrochemical biosensing, laser-induced graphene, gas sensing, or intelligent packaging in general. In contrast, the present review specifically focuses on graphene-based sensors for food freshness and spoilage monitoring and provides a comparative analysis across different sensing mechanisms, including colorimetric, electrical/chemiresistive, optical/fluorescence, QCM, and photoelectrochemical approaches. Particular attention is also given to aspects that have received limited systematic discussion in previous reviews, including performance under realistic storage conditions, humidity and temperature interference, sensor fouling and long-term stability, machine-learning-assisted data analysis, and commercialization readiness. By integrating analytical performance with practical implementation criteria, this review aims to identify the main technological gaps and the graphene-based sensing strategies with the greatest potential for real-world food freshness monitoring.

## 2. Graphene-Based Sensing Strategies for Food Freshness Monitoring

**Figure 3:**
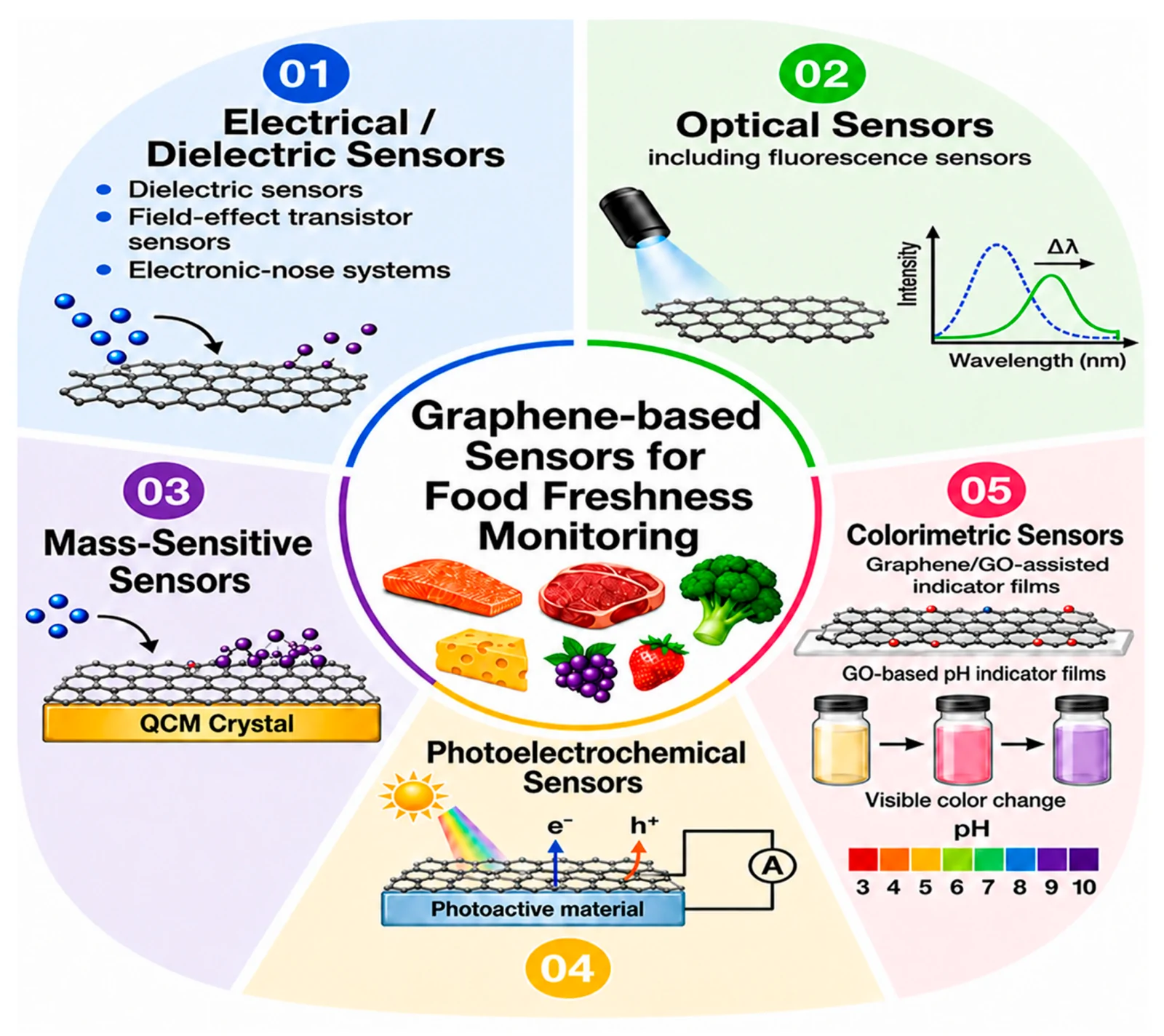
Schematic classification of graphene-based sensors for food freshness monitoring, including electrical/dielectric, optical, mass-sensitive, photoelectrochemical, and colorimetric sensors.

Graphene-based food freshness sensors can be classified according to their dominant sensing mechanism, including electrical/dielectric, field-effect, resistance-based electronic-nose, optical/fluorescence-based, photoelectrochemical, mass-sensitive, and colorimetric approaches (see Figure 3). In the following section, representative examples are discussed.

### 2.1. Electrical/Dielectric Sensors

Dielectric sensors represent one route for non-destructive freshness assessment. One representative example was reported by Li et al. [23], who developed a sensing platform based on graphene-coated copper film electrodes fabricated by chemical vapor deposition [24]. In this case, graphene was used to improve the chemical stability and corrosion resistance of the electrodes, enabling their application in non-contact dielectric spectroscopy measurements. Rather than relying on destructive analysis or chemical reagents, meat freshness was evaluated by tracking changes in dielectric properties. This is an important aspect, since it reflects a shift away from conventional laboratory-based testing toward non-invasive and in situ monitoring approaches. The dielectric data collected from the samples were further processed using a machine-learning model based on XGBoost, which achieved a classification accuracy of up to 96.36% in distinguishing freshness levels.

Field-effect transistor (FET)-based sensors [25] operate by modulating the current flowing between the source and drain electrodes through an electric field applied at the gate. In graphene-based FETs, graphene acts as the conductive channel and is highly sensitive to changes occurring at its surface. When target molecules or spoilage-related compounds interact with the graphene channel or with a functional layer deposited on it, they can induce charge transfer, electrostatic gating, or local changes in surface potential. These effects modify the carrier concentration in graphene and are detected as variations in the source–drain current, shifts in the threshold voltage, or changes in the Dirac point. Therefore, graphene field-effect transistor (GFET) sensors are particularly suitable for label-free and real-time detection, because molecular interactions at the surface are directly converted into an electrical signal. Saiki et al. [26] used a GFET to discriminate between fresh and expired *Agaricus bisporus* mushrooms. In this study, the electrical response of the GFET clearly differed depending on sample condition, with variations in current spanning nearly one order of magnitude between fresh and degraded mushrooms. The sensing behavior was attributed to the particular architecture of the device, in which the graphene channel was designed to interact directly with a titanium dioxide surface. These interactions promoted protonation and deprotonation processes, generating charge transfer phenomena that modulated the electrical output of the sensor according to the condition of the food sample. Importantly, the electrical sensing results were supported by additional analytical methods. Fourier-transform infrared spectroscopy (FTIR) revealed subtle differences in functional groups, especially in spectral regions associated with proteins and carbohydrates, while ultra-high-resolution mass spectrometry enabled the identification of spoilage-related molecular markers, including sugars, peptides, nucleotides, and microbial by-products.

Electronic-nose systems are bio-inspired sensing platforms designed to mimic the human olfactory system for the detection and classification of volatile compounds released from food [27]. They are based on an array of broadly responsive gas sensors that interact with odor molecules and generate characteristic signal patterns, often referred to as volatile fingerprints. These signals are then processed using multivariate statistical or machine-learning methods, such as PCA, LDA, SVM, or artificial neural networks, to classify odor profiles and distinguish freshness stages, spoilage, contamination, or adulteration. In food freshness monitoring, e-nose systems are particularly useful because spoilage processes generate volatile organic compounds, including ammonia, trimethylamine, sulfur compounds, and other VOCs, which can be detected rapidly and non-destructively. Depending on the sensor array, the response may arise from changes in electrical properties, mass, conductivity, or other sensor outputs after exposure to volatile compounds.

**Figure 4:**
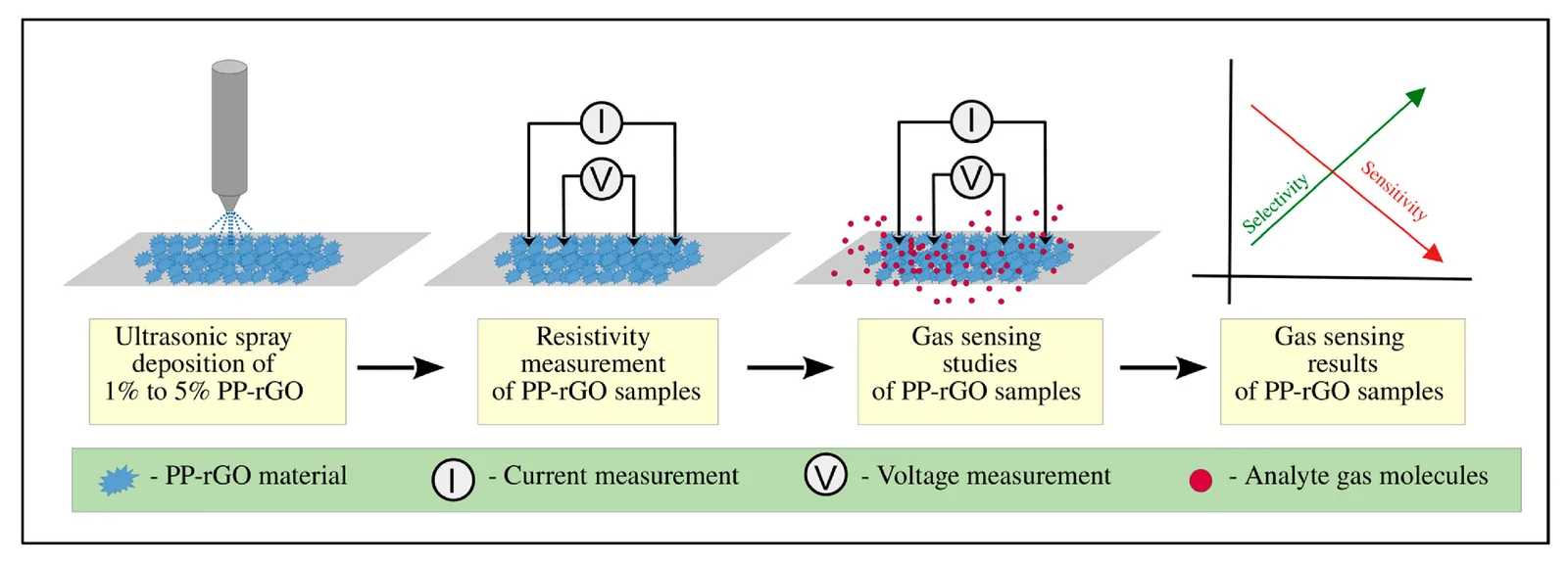
Scheme of PP-rGO gas sensor. Reproduced from Ref. [28].

Nadekar et al. [28] (see Figure 4) developed an electronic-nose-type sensing platform based on a composite combining polyvinyl alcohol (PVA), polyethylene glycol (PEG) and reduced graphene oxide (rGO) [29]. In this system, the polymer matrix provides flexibility and moisture responsiveness, while rGO contributes electrical conductivity and enhanced sensing performance. The composite was prepared through a low-temperature embedding process, an approach that helped preserve the integrity of the sensitive components and avoid thermal degradation during fabrication. The resulting sensor exhibited high sensitivity toward volatile amines, which are well-known by-products of fish spoilage. The sensing mechanism relied on variations in the electrical resistance of the rGO network induced by interactions between the polymer/graphene composite and gaseous spoilage products. These resistance changes were found to correlate with different stages of fish freshness, indicating the suitability of the system for real-time monitoring.

### 2.2. Optical Sensors

Optical sensors [30] are widely investigated in food safety monitoring because they can provide rapid, sensitive, portable, and non-destructive detection of chemical and biological changes in food systems. These platforms convert interactions between the target analyte and the sensing material into measurable optical signals, including fluorescence variation, refractive-index changes, Raman scattering enhancement, elemental emission, or visible color changes. Among these approaches, fluorescence-based sensors are particularly relevant because quantum dots and carbon-based nanomaterials can offer high sensitivity, good photostability, and rapid response. In quantum dot fluorescence sensors, target molecules may induce fluorescence quenching, fluorescence recovery, or ratiometric signal changes, for example through mechanisms such as inner-filter effects or analyte-induced modulation of the emissive probe. These optical responses can then be correlated with contaminant or spoilage-marker concentration, supporting on-site and real-time food monitoring.

Zhang et al. [31] developed a fluorescent sensing platform based on nitrogen-doped graphene quantum dots (N-GQDs) supported on halloysite nanotubes and embedded in a PVA matrix. In this case, the sensing target was ammonia, one of the most important volatile compounds associated with fish spoilage. The incorporation of N-GQDs onto halloysite nanotubes improved fluorescence performance, mainly by reducing aggregation through electrostatic interactions and hydrogen bonding. The hybrid film exhibited a sensitive fluorescence response toward ammonia, and the sensing mechanism was attributed to fluorescence quenching induced by photoinduced electron transfer processes. The response was linear over a broad concentration range and showed a low detection limit, demonstrating the effectiveness of the design. From an application point of view, the system was tested in the monitoring of both seawater and freshwater fish stored under different temperature conditions, and the changes in fluorescence intensity were found to correlate with spoilage progression.

**Figure 5:**
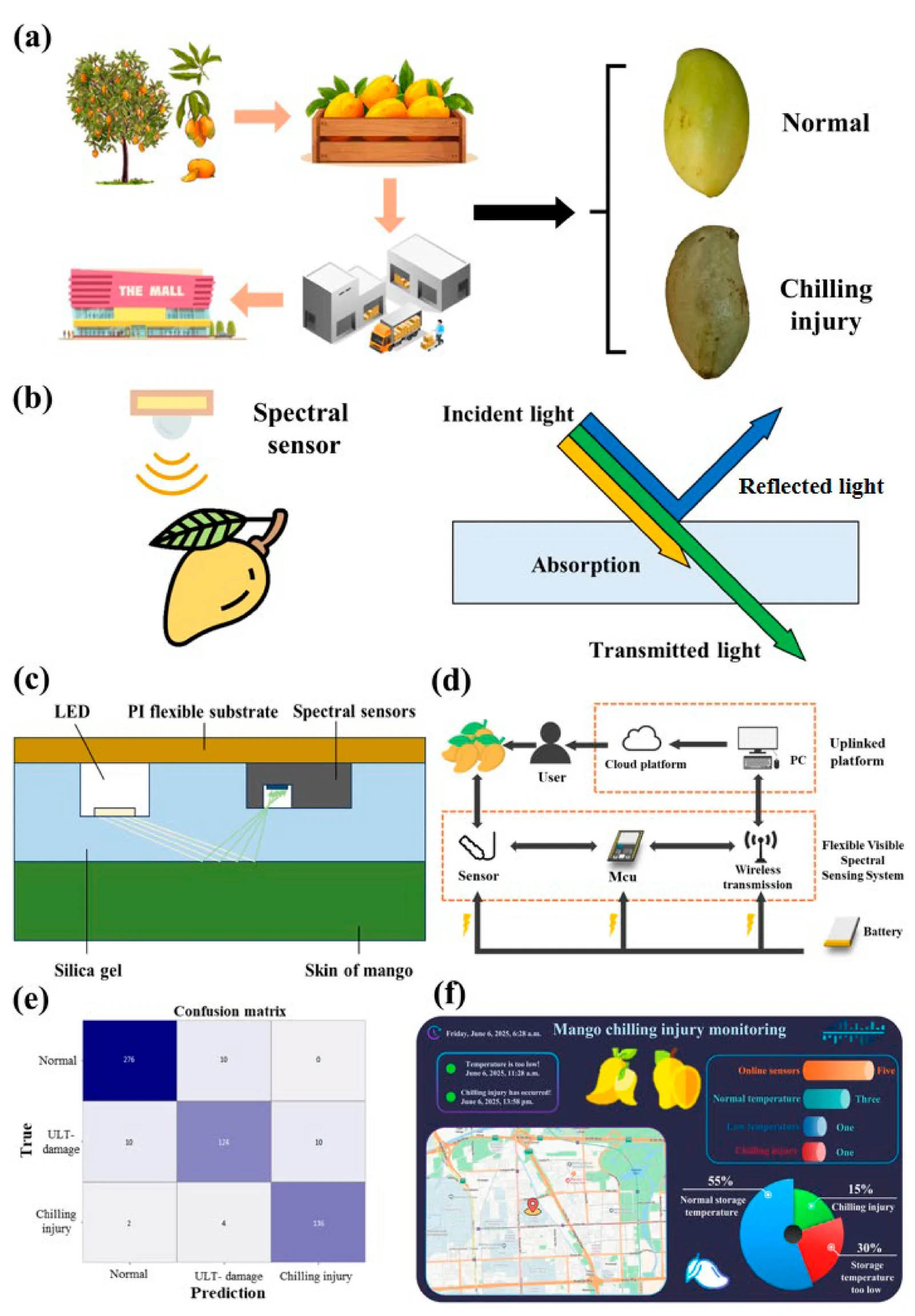
System-level overview of the mango freshness monitoring platform: (**a**) supply-chain workflow from harvesting to consumption; (**b**,**c**) spectral sensing principle; (**d**) sensing-system logic; (**e**) prediction-model confusion matrix; and (**f**) cloud monitoring dashboard. Reproduced from Ref. [32].

Ma et al. [32] (see Figure 5) developed a flexible sensing system based on visible light spectroscopy for the real-time detection of chilling injury in mangoes during cold storage. The sensing platform was fabricated using laser-induced graphene (LIG) combined with electroplated copper and laser etching techniques, resulting in a compact and flexible device. The system integrates sensing, data acquisition, and processing within a perception–analysis–warning framework, enabling continuous monitoring. Using *Yunnan Yumang* mangoes as a model system, the authors constructed a dataset with different levels of chilling injury. After data preprocessing, including Z-score normalization, machine-learning analysis based on an SVM achieved high prediction accuracy, up to 95.5%, in classifying damage levels. The system also exhibited low power consumption, long operational lifetime, and stable signal transmission.

### 2.3. Photoelectrochemical Sensors

Photoelectrochemical (PEC) sensing has emerged as a promising analytical approach for food-contaminant monitoring because it combines high sensitivity, good selectivity, low cost, simple operation, and rapid response [33]. In a typical PEC system, a photoactive material is irradiated by a light source, generating electron–hole pairs. Their separation and migration toward the interface lead to redox reactions and produce a measurable photocurrent, which is used as the analytical signal. The recognition of a target analyte modifies the physicochemical environment of the photoelectrode or electrolyte, affecting charge separation, interfacial transfer, or surface redox processes. As a result, variations in photocurrent can be correlated with analyte concentration. Since the excitation source is optical while the output signal is electrical, PEC sensing benefits from reduced background noise compared with conventional electrochemical methods. Moreover, the possibility of using different photoactive materials and amplification strategies makes this technique a versatile platform for the rapid and quantitative detection of a wide range of food-related contaminants. Wang et al. [34] developed a photoelectrochemical biosensor for the indirect detection of lipase activity in rice bran, using glycerol as the reaction marker. In this work, the sensing platform was based on a titanium dioxide (TiO_2_) nanofiber/rGO nanocomposite prepared by electrospinning. The presence of rGO enhanced the photoelectric properties of the system, contributing to the analytical performance of the biosensor. After immobilization of glycerol dehydrogenase, the device enabled the quantification of glycerol over a broad concentration range, with a linear relationship between the photoelectrochemical signal and glycerol content. Since glycerol is generated through lipase-induced hydrolysis of lipids, the sensor provided an indirect but effective route for monitoring a key process involved in grain spoilage and rancidity. The device showed low detection limits, good reproducibility over repeated uses, satisfactory long-term stability, and minimal interference from other species, indicating good selectivity.

### 2.4. Mass-Sensitive Gas Sensor Arrays

Mass-sensitive gas sensors rely on the adsorption or interaction of gas molecules with a sensing layer deposited on the sensor surface. This interaction produces a small mass change, or a related variation in a physical property of the device, which is then converted into a measurable electrical signal. In this way, information on gas concentration or composition can be obtained. Among mass-sensitive platforms, quartz crystal microbalance (QCM) and surface acoustic wave (SAW) sensors are widely used, since both can detect very small mass variations associated with analyte adsorption [35]. An important contribution in this area was reported by Chen et al. [36], who developed a gas sensor array based on quartz crystal microbalance technology (QCM) for the monitoring of VOCs released during salmon spoilage. In this system, GO was employed as one of the principal sensing materials together with other carbon-based nanostructures. GO played a central role because of its high specific surface area and the presence of oxygen-containing functional groups, both of which favor adsorption of volatile compounds such as ammonia, trimethylamine, dimethylamine, and formaldehyde. These compounds are strongly associated with degradation processes in protein-rich foods and therefore represent useful freshness markers. The QCM sensors functionalized with GO and the other nanomaterials exhibited good sensitivity, selectivity, and repeatability toward the target analytes. When applied to salmon meat, the sensor array enabled discrimination among different freshness stages on the basis of volatile emission patterns. The response data were further analyzed by multivariate approaches, including principal component analysis and support vector machine (SVM) classification, and the obtained classifications were validated by correlation with conventional spoilage indicators such as total volatile basic nitrogen. This study shows how GO can be effectively integrated into more complex sensor array architectures for non-destructive, real-time food freshness monitoring.

### 2.5. Colorimetric Sensors

Colorimetric sensors provide visual information on food freshness through color changes induced by spoilage-related chemical or environmental variations [37]. In intelligent packaging, these systems typically rely on pH- or gas-responsive dyes, such as anthocyanins, curcumin, betalains, or other halochromic pigments, embedded in polymeric or biopolymeric matrices. During food degradation, microbial and enzymatic processes generate volatile amines, ammonia, organic acids, CO_2_, H_2_S, or other metabolites that can modify the local pH, redox environment, or gas composition around the indicator. These changes alter the molecular structure or electronic state of the dye, producing a visible color transition that can be correlated with freshness parameters such as pH, TVB-N, microbial growth, or volatile-marker accumulation. However, indicators based on pH-responsive water- or alcohol-soluble pigments generally provide only semi-quantitative information and often suffer from limited sensitivity, which remains a critical limitation for practical freshness monitoring [38].

A large number of studies have focused on the development of graphene-based intelligent packaging systems capable of providing real-time visual feedback on food freshness [39]. In graphene-based systems, GO, rGO, or GNPs usually act as functional additives that improve mechanical strength, barrier properties, UV protection, dye stability, antimicrobial activity, and interfacial interactions within the sensing film.

Huang et al. [40] reported a hybrid film based on carboxymethyl cellulose and chitosan, incorporating anthocyanins as natural pH-sensitive indicators and graphene nanoplatelets (GNPs) as functional additives. The integration of chitosan and GNPs enhanced the physicochemical and mechanical properties of the films, as well as their ultraviolet (UV) resistance and antibacterial performance. The colorimetric component exhibited a pronounced response over a wide pH range, from 2 to 12, with a visible color transition from red to yellow, enabling direct visual assessment of food freshness without the need for analytical instrumentation. When applied to shrimp preservation, the films showed a clear correlation between color variation and spoilage indicators such as pH and total volatile basic nitrogen. At the same time, the presence of GNPs and chitosan contributed to improved preservation performance, confirming their role in active packaging systems.

A similar strategy was proposed by Chowdhury et al. [41], who developed a multifunctional film based on fish-scale-derived gelatin, reinforced with biosynthesized GO and incorporating anthocyanins as pH-responsive indicators. In this system, gelatin obtained from fish processing waste served as the main biopolymer matrix, while GO was introduced to overcome intrinsic limitations such as poor mechanical strength and barrier properties. The addition of GO and anthocyanins improved tensile strength, thermal stability, water vapor resistance, and UV-blocking ability, indicating effective dispersion within the polymer matrix. The films also exhibited rapid biodegradability under soil conditions. From a sensing perspective, the anthocyanins enabled a clear pH-dependent colorimetric response during fish storage, with strong correlations observed between color variation and conventional spoilage indicators such as pH and total volatile basic nitrogen.

Prasetya et al. [10] developed a chitosan-based halochromic film reinforced with GO and incorporating red cabbage extract as a natural pH-sensitive indicator. The incorporation of GO significantly improved mechanical strength, surface hydrophobicity, and antibacterial activity, with the formulation containing 0.75% GO showing the best performance. Thermal analyses confirmed an enhancement in thermal stability upon GO addition, indicating effective interaction between the filler and the polymer matrix. The films displayed a pronounced pH-dependent colorimetric response and enabled visual detection of spoilage processes. When applied to chicken meat, a clear indication of degradation was observed within 48 h of storage. In addition to their functional performance, these films were based on biodegradable and food-compatible components.

Kafashan et al. [42] developed a composite film based on basil seed gum incorporating Malva sylvestris extract as a natural dye and GO as a reinforcing agent. The addition of the extract provided a pronounced colorimetric response over a wide pH range, enabling visual detection of spoilage-related changes. However, the extract alone negatively affected the mechanical and thermal stability of the polymer matrix. The introduction of GO at an optimized concentration improved these properties, enhancing both mechanical strength and thermal resistance. Release studies showed more controlled behavior in GO-containing films, indicating improved structural stability. When applied to salmon storage, the color variation correlated well with pH and total volatile basic nitrogen, confirming the effectiveness of the system as a freshness indicator.

A related system was reported again by Kafashan et al. [43], based on soluble soybean polysaccharide films incorporating grape skin anthocyanin extract and GO. The anthocyanin extract provided pH sensitivity and enabled visible color changes in response to environmental variations associated with spoilage. However, its incorporation alone negatively affected mechanical and thermal properties and increased moisture content and water vapor permeability. The addition of GO mitigated these effects, improving mechanical strength, thermal stability, and reducing moisture uptake and permeability. When applied to salmon packaging, the films exhibited clear color variations correlated with spoilage indicators such as pH and total volatile basic nitrogen.

Fathi et al. [44] reported the fabrication of a locust-bean-gum-based film incorporating anthocyanins extracted from Viola as a natural colorimetric indicator together with GO. The presence of anthocyanins enabled pH-responsive behavior, while the addition of GO improved mechanical properties and thermal stability. GO-containing films also exhibited improved moisture-related properties, including reduced water sensitivity and enhanced surface characteristics, while maintaining good biocompatibility. Release studies indicated controlled release of anthocyanins over time, supporting sustained sensing functionality. In addition, the incorporation of the extract contributed to enhanced antioxidant activity, which may be beneficial for food preservation.

Zhao et al. [45] developed composite films based on carboxymethyl cellulose, anthocyanins, and GO. The incorporation of GO significantly improved mechanical strength and barrier properties against oxygen and water vapor, due to strong interactions between the polymer and the graphene-based filler. The films exhibited clear color changes in response to pH variations and exposure to ammonia, a key indicator of protein degradation. The colorimetric response showed strong correlation with spoilage parameters such as pH and total volatile basic nitrogen. In addition, the films demonstrated good biodegradability, supporting their application in sustainable packaging.

### 2.6. Environmental and Multifunctional Monitoring Systems

In addition to direct sensing of spoilage markers and colorimetric intelligent films, another important research direction involves the monitoring of environmental parameters that influence food degradation. In these approaches, graphene-based materials are used to detect variations in temperature, humidity, oxygen levels, or other external conditions that are closely related to food quality and shelf life.

Humidity-responsive hydrogel sensors have recently been investigated as multifunctional systems for food preservation and environmental monitoring. In this context, Han et al. [46] developed a porous hydrogel based on acrylic acid and bagasse-derived cellulose, incorporating GO and citral to achieve both sensing and antibacterial functionalities.

The hydrogel was fabricated using a cold-plasma-assisted approach, which promoted the effective incorporation of GO and citral through hydrogen-bond interactions within the polymer network. The addition of GO enhanced the electrical conductivity and humidity sensitivity of the system, with optimized compositions showing high response under controlled testing conditions. The sensor also exhibited stable and reversible behavior during cyclic humidity variations, indicating good durability and repeatability.

In addition to its sensing capabilities, the incorporation of citral conferred antibacterial activity against common pathogens such as *Escherichia coli* and *Staphylococcus aureus*. This multifunctional behavior was further demonstrated in fruit preservation tests, where the hydrogel system contributed to extending the shelf life of mangoes. The sensing mechanism was associated with changes in water absorption and the controlled release of citral, which could also be visually monitored through variations in color.

Humidity is an additional important parameter associated with food spoilage, especially in packaged products. Shboul et al. [47] developed a flexible humidity sensor based on a gelatin-modified graphene ink designed for real-time detection of environmental changes. The composite combines the electrical conductivity of graphene with the hygroscopic nature of gelatin, enabling a sensitive response to variations in relative humidity. The sensor operates over a broad humidity range and exhibits stable, repeatable behavior with low hysteresis and good mechanical flexibility. When tested in food packaging environments, the device successfully detected humidity changes related to spoilage phenomena in meat products. The sensing mechanism is associated with moisture accumulation during degradation, allowing indirect monitoring of freshness without direct contact with the food. Recent developments further demonstrate the potential of graphene-based materials for flexible humidity monitoring. Lu et al. [48] developed a self-healable graphene–cellulose nanofibril composite exhibiting simultaneous strain and humidity responsivity.

Temperature control is another key factor in food preservation, particularly for preventing frost damage. In this context, Chen et al. [49] proposed a flexible sensing and heating system based on LIG for temperature monitoring and frost protection. The developed system integrates a flexible temperature sensor with a graphene-based heating element. The performance of the heating patches was evaluated by analyzing electrical, thermoelectric, and morphological properties, identifying optimal fabrication parameters. The functionality of the system was demonstrated through simulated applications on fruits such as oranges and pears, where the heating element enabled rapid temperature recovery after exposure to cold stress conditions. At the same time, the integrated sensor allowed continuous monitoring of surface temperature.

Monitoring of oxygen levels is also critical, particularly in modified atmosphere packaging systems. In this context, Huang et al. [50] developed a visual colorimetric oxygen indicator based on a graphene/TiO_2_ composite integrated within a polymeric matrix. The sensing mechanism relies on the oxygen-dependent redox behavior of methylene blue, which produces a visible color change in response to oxygen presence. The graphene/TiO_2_ composite improves the stability and performance of the system, enabling nondestructive and real-time assessment of oxygen ingress. The response follows pseudo-first-order kinetics, and the reaction rate can be tuned by adjusting dye concentration, allowing control over the recovery behavior of the indicator.

Recent research has increasingly moved toward the development of integrated and multifunctional sensing platforms. These systems are designed to simultaneously monitor multiple variables associated with food spoilage, while also enabling advanced functionalities such as wireless communication and real-time data accessibility. Jung et al. [51] introduced a laser-induced paper sensor (LIPS) based on a hybrid structure of LIG integrated onto paper substrates. The fabrication process involves direct laser irradiation of commercial paper, which converts the surface into a conductive and porous graphene-based structure. This configuration enables both chemical and thermal sensing capabilities, allowing the detection of parameters relevant to food spoilage, such as gas concentration and temperature. The resulting sensors exhibit stable electrical properties and sensitivity to environmental variations, supporting real-time monitoring. As a proof of concept, the system was applied directly to food packaging, where it enabled simultaneous temperature tracking and detection of chemical changes associated with degradation. The collected data could be transmitted wirelessly to external devices, facilitating continuous and user-accessible monitoring. Recent advances in laser-induced graphene also suggest possible routes toward self-powered sensing architectures. Liu et al. [52] developed a laser-induced graphene/ITO heterostructure capable of self-powered ion sensing through the ionovoltaic effect.

Another advanced multifunctional system was reported by Kurina et al. [53], who developed a dual-mode ammonia sensor combining colorimetric and resistive sensing functionalities. The system is based on a paper-supported film incorporating anthocyanins as a natural pH-sensitive indicator, reinforced with polyvinyl alcohol, and containing GNPs to provide electrical conductivity. The sensor exhibited high sensitivity toward ammonia, a key volatile compound associated with protein degradation. The combination of colorimetric and electrical responses enables more robust detection of spoilage. Moreover, the integration of near-field communication technology (NFC) allows wireless and battery-free signal transmission, supporting real-time and user-friendly monitoring. The fabrication process, based on low-temperature coating techniques, also supports scalability and cost-effectiveness. The practical applicability was demonstrated in shrimp storage, where both colorimetric and electrical signals correlated with ammonia release.

## 3. Comparative Overview of Graphene-Based Sensing Strategies and Key Performance Indicators

To provide a clearer comparative overview of the reviewed literature, Table 1 summarizes the main graphene-based sensing strategies proposed for food freshness monitoring. In addition to the qualitative comparison reported in Table 1, the analytical performance of these sensing systems is summarized in Table 2. Particular attention is given to key performance indicators (KPIs) relevant to food freshness sensing, including sensitivity or detection range, limit of detection when available, selectivity, response and recovery times, reproducibility, calibration, and stability. These parameters are essential to evaluate whether a sensing platform can be translated from laboratory demonstrations to practical food-packaging or real-time monitoring applications.

Table 1 highlights a clear distinction between platforms designed for direct detection of spoilage-related analytes and systems that indirectly assess food quality through environmental, optical, or physicochemical changes. Electrical, fluorescence, PEC, and QCM platforms generally provide analyte-specific or quantitative information, whereas colorimetric and environmental sensors favor simple integration into packaging and real-time visual or condition-based monitoring. Across the reviewed studies, however, validation is still frequently restricted to a single food matrix, indicating that cross-matrix robustness and large-scale practical validation remain important gaps.

A quantitative comparison of the reported analytical figures of merit in Table 2 reveals some general differences among the sensing platforms, although direct ranking is limited by the use of different analytes, concentration ranges, and experimental conditions. Among gas-sensing systems, response times ranged from 51.5 s for the multifunctional LIG sensor [51] to 310–440 s for the PVA–PEG/rGO chemiresistive sensor [28], while the QCM platform [36] showed recovery times of 90–120 s, compared with 65.9 s for [51] and 30–70 s for [28]. These values indicate substantial differences in response dynamics, although they should be regarded as indicative rather than directly equivalent because different gases and concentrations were tested.

Calibration linearity was generally high across the quantitative platforms, with reported R^2^ values ranging from 0.94 to 0.99 for the QCM array [36], 0.98 for the fluorescence sensor [31], 0.99 for the chemiresistive sensor [28], and approximately 0.996–0.998 for the PEC sensor [34]. Thus, most platforms showed strong calibration performance, although the values refer to different target analytes and cannot be used alone to establish analytical superiority.

Overall, the available quantitative data reported in Table 2 suggest trade-offs between response dynamics, calibration performance, and long-term stability.

## 4. Machine-Learning-Assisted Graphene-Based Sensors for Food Freshness Monitoring

Graphene-based materials are promising platforms for food freshness and spoilage monitoring, particularly when combined with machine-learning (ML) methods such as Support Vector Machines (SVMs) and XGBoost. ML algorithms can further improve sensor performance by analysing complex and multidimensional response patterns, supporting rapid classification and prediction of spoilage events [54]. Capman et al. [54] demonstrated the potential of supervised ML for rapid analysis of graphene-based sensor arrays, achieving 98% accuracy in the classification of five VOCs at different concentrations using a Bootstrap Aggregated Random Forest model. However, accuracy decreased to 89% when a structurally similar analyte was introduced, highlighting the importance of dataset complexity, analyte similarity, noise, and algorithm selection for model robustness and generalizability.

The effectiveness of ML-assisted graphene sensors strongly depends on data quality and availability. Algorithms such as SVM and XGBoost require sufficiently representative training datasets, and limited or poorly distributed data can reduce prediction accuracy and model generalizability [55]. More generally, although not specifically focused on graphene-based materials, Han et al. [55] highlighted that SVM is particularly suitable for nonlinear freshness-related datasets, but its performance is sensitive to kernel selection, parameter tuning, noise, and overlapping data. Reliable ML analysis also requires appropriate preprocessing—including noise reduction, baseline correction, normalization, and feature selection—and rigorous validation. Training, validation, and test datasets should be properly separated, while cross-validation can be used to assess generalizability and reduce the risk of overfitting. These considerations are particularly relevant to graphene-based food sensors, where variations in sensor response, food matrix, storage conditions, and environmental parameters may affect model robustness. In this context, Pannone et al. [56] demonstrated that ML applied to graphene-based ISFET arrays can improve classification and quantification while mitigating cycle-to-cycle, sensor-to-sensor, and chip-to-chip variability. The approach successfully discriminated food spoilage, fraud, and safety-related conditions, highlighting the potential of ML to improve the robustness and practical reliability of graphene-based food sensors. In contrast to the promising aspects of ML models, their complexity can hinder interpretability and generalizability across different food types. Addressing these challenges through improved data collection and model refinement will be essential for the broader adoption of these technologies in food safety monitoring.

## 5. Commercialization Readiness of Graphene-Based Sensing Technologies

A critical comparison of graphene-based sensing strategies for food freshness monitoring reveals a clear trade-off between analytical performance and practical applicability. Colorimetric, chemiresistive/electrical, fluorescence/optical, quartz crystal microbalance (QCM), and photoelectrochemical sensors provide different advantages in terms of sensitivity, response time, instrumentation, fabrication complexity, and suitability for real-world food monitoring. Therefore, the most sensitive sensing approach is not necessarily the most appropriate for practical implementation, particularly when low cost, simple operation, and integration into food packaging are required.

Colorimetric sensors currently represent one of the most practical approaches for intelligent food packaging, even though their analytical sensitivity can be lower than that of electrical or optical sensors. For instance, colorimetric GO-based indicators appear particularly promising for commercialization because they provide visual readout and can be incorporated into flexible polymeric or biopolymer films. GO/anthocyanin films have already been validated for monitoring real food spoilage, showing correlations between colour changes and conventional freshness indicators such as pH [42]. Their rapid response and successful validation with different food matrices support their potential for practical freshness monitoring.

In contrast, chemiresistive and other electrical graphene-based sensors generally provide higher sensitivity and faster quantitative detection. Changes in electrical resistance, conductance, impedance, or field-effect characteristics can enable the detection of low concentrations of spoilage-related compounds. Electrical measurements can also be readily digitized, facilitating data processing and integration with ML algorithms. For example, Xia et al. [57] developed a LIG/rGO patch for fish freshness monitoring with a reported cost below $1, high impedance reproducibility, and operation under refrigerated and high-humidity conditions.

This makes these platforms particularly attractive for continuous or automated food-quality monitoring. However, their practical implementation is generally more demanding than that of colorimetric indicators because electronic components, power sources, calibration procedures, and dedicated readout systems may be required. Sensor drift and variations caused by temperature, humidity, and device-to-device differences can also compromise long-term reliability. Thus, electrical sensors may provide superior analytical performance but at the expense of greater system complexity and potentially higher cost.

Fluorescence and optical sensors offer another highly sensitive strategy and can provide selective detection of specific spoilage markers [58]. Their optical response may enable low detection limits and effective discrimination of target analytes. However, fluorescence-based systems generally require excitation sources, photodetectors, filters, or imaging devices, which increase instrumentation requirements and limit their direct integration into inexpensive disposable food-monitoring systems.

QCM sensors provide very high sensitivity to mass changes occurring on the sensing surface and can be effectively functionalized for the detection of volatile compounds associated with food deterioration [59]. Nevertheless, their practical use is limited by the rigid quartz substrate and the requirement for dedicated frequency-measurement electronics.

Finally, photoelectrochemical sensors combine electrochemical detection with photoactive materials and can provide high sensitivity and selectivity [59]. However, their multi-component architecture and requirement for light excitation and electrochemical readout increase fabrication and operational complexity.

## 6. Food Safety and Regulatory Considerations

The use of graphene and graphene-related materials in food-contact applications also raises important safety and regulatory concerns, particularly regarding the possible migration of nanomaterials from packaging into food. Migration is strongly influenced by the characteristics of the degree of nanomaterial incorporation and the temperature and composition of the contacting medium. Experimental evidence indicates that graphene nanoplatelets can be released from polymer nanocomposites under specific conditions. For example, Velichkova et al. [60] observed the release of graphene nanoplatelets from PLA-based composite films during migration tests at elevated temperature, which was associated with swelling and hydrolytic degradation of the polymer matrix. More recent results by Khajavi et al. [61] have also shown that matrix formulation can significantly influence graphene release: the incorporation of toughening agents and compatibilizers into PLA/graphene nanocomposites reduced polymer swelling, degradation, and graphene migration into food simulants.

Toxicological assessment is equally important because any migrated graphene-related material may potentially result in oral exposure. Available studies do not provide a simple conclusion regarding safety. For instance, Guarnieri et al. [62] investigated few-layer graphene and GO under simulated gastrointestinal digestion and found no detectable impairment of intestinal barrier integrity, inflammation, or cytotoxicity in their in vitro model after chronic exposure; however, the materials remained biopersistent and the authors noted that possible long-term adverse effects could not be excluded. Therefore, future safety assessments should consider not only the nominal graphene composition but also particle size, aggregation state, surface chemistry, dose, transformation during digestion, and realistic exposure levels.

From a regulatory perspective, graphene-based sensors for food freshness monitoring must be evaluated according to their intended configuration and potential exposure of the food to the sensing material. In the European Union, sensors incorporated into food-contact materials or intelligent food-monitoring systems may fall within the general framework established by Regulation (EC) No 1935/2004, which requires that materials do not transfer their constituents to food at levels that could endanger human health or cause unacceptable changes in food composition or organoleptic properties. Intelligent materials and articles designed to monitor the condition of packaged food or its surrounding environment are additionally addressed by Regulation (EC) No 450/2009. Particular attention is required when graphene or its derivatives are present in nanoform, since regulatory assessment must consider the possibility of migration and consumer exposure. The European Food Safety Authority (EFSA) provides specific guidance for the physicochemical characterization and risk assessment of nanomaterials and small particles relevant to food and food-contact applications. Consequently, the practical implementation of graphene-based food freshness sensors cannot be assessed solely on the basis of sensing performance, but should also consider the direct or indirect contact with food, potential migration, exposure conditions, and material-specific toxicological safety.

Consumer acceptance represents a further consideration for commercial implementation. Studies on food nanotechnology indicate that willingness to purchase nano-enabled food products is influenced by the perceived balance between benefits and risks and trust in the food industry [63]. Overall, standardized migration testing, nanospecific toxicological assessment, clear regulatory pathways, and effective risk communication will be essential for the safe commercialization and public acceptance of graphene-based food sensor technologies.

## 7. Real-World Application Challenges and Future Research Directions

Despite the excellent sensitivity and selectivity frequently reported under controlled laboratory conditions, the translation of graphene-based sensors to real-world food monitoring remains challenging. In practical applications, variations in relative humidity and temperature can significantly affect adsorption/desorption processes and consequently modify the electrical response of graphene-based sensing layers, potentially introducing baseline fluctuations and signal drift. Recent studies have started to evaluate graphene-based sensing platforms under conditions closer to real food-storage environments. For example, Xia et al. [57] developed a conformal temperature/impedance sensing patch based on laser-induced graphene (LIG) and reduced graphene oxide (rGO) that was directly attached to refrigerated fish. The device enabled continuous low-temperature monitoring for 120 min without significant interference from high humidity and achieved a freshness classification accuracy of 93.07% using a support vector machine model (SVM). The reported cost of less than $1 per patch also highlights the potential of such systems for practical and scalable food-quality monitoring.

Similarly, Yang et al. [64] proposed a temperature- and humidity-compensation approach combined with a Long Short-Term Memory (LSTM) model to improve the accuracy of ammonia concentration measurements obtained using graphene sensors under changing environmental conditions, highlighting the potential of data-driven correction strategies for reducing environmental interference.

Interestingly, some of these challenges have already been addressed in food-freshness sensors based on alternative sensing materials. Tang et al. [65], for example, developed a metal-oxide sensor array capable of monitoring different foods and food mixtures under refrigerated conditions for several days. The system was evaluated under different relative humidity levels and integrated with humidity sensing, data processing, and wireless communication. However, the authors still identified environmental fluctuations and signal drift as important issues requiring compensation strategies. These results provide a useful benchmark for the future development of graphene-based sensors under realistic cold-chain conditions.

Lv et al. [66] developed a wireless ammonia sensor with self-humidity compensation for real-time food freshness monitoring. The system was evaluated with real food samples and represents a useful benchmark for the development of graphene-based sensors operating under variable humidity conditions. Similarly, Zhu et al. [67] reported a flexible and porous polymer-based sensor for selective room-temperature ammonia detection with potential applications in food monitoring.

Complex food matrices introduce additional challenges because proteins, lipids, salts, moisture, and other components can adsorb onto the sensing surface, causing fouling, cross-sensitivity, loss of active sites, and progressive deterioration of the analytical response. Long-term stability, reproducibility between devices, calibration drift, mechanical durability, and sensor regeneration therefore remain insufficiently investigated compared with short-term laboratory performance. Future research should consequently prioritize standardized testing under realistic storage and cold-chain conditions, prolonged stability and repeated-use experiments, systematic evaluation of fouling and interfering species, and the development of protective or antifouling interfaces that preserve analyte accessibility. The integration of temperature/humidity compensation, sensor arrays, machine-learning-based drift correction, wireless readout, and smart packaging represents another important direction toward robust and autonomous monitoring. Ultimately, industrial implementation will require validation with real food samples over commercially relevant storage periods, together with scalable fabrication, batch-to-batch reproducibility, low power consumption, and cost-effective integration into existing packaging and food-distribution systems.

Despite these recent advances, fully integrated multifunctional sensing platforms remain relatively scarce. While individual approaches successfully target specific parameters such as pH changes, volatile compounds, temperature, or humidity, only a few studies combine these functionalities into unified systems. Safety and regulatory considerations represent an additional barrier to commercialization and are discussed in detail in Section 6.

Finally, for colorimetric intelligent packaging systems, the stability of natural pigment-based indicators remains a specific limitation. Although anthocyanins and other plant-derived dyes provide effective pH sensitivity, they are susceptible to degradation under environmental conditions such as light, moisture, and temperature. This can reduce the reliability of visual indicators over time. Future research should explore stabilization strategies, including encapsulation techniques and hybrid systems with graphene-based materials, to improve durability while maintaining sensitivity.

## 8. Conclusions

Graphene-based materials offer versatile strategies for food freshness monitoring, including electrical, optical, photoelectrochemical, QCM, colorimetric, and multifunctional sensing platforms. Overall, the reviewed studies indicate that no single sensing strategy simultaneously maximizes analytical performance, simplicity, robustness, and packaging compatibility.

Electrical and optical systems generally offer higher quantitative performance and are particularly suitable for continuous or automated monitoring, although their practical implementation may require dedicated instrumentation, calibration, and compensation for environmental effects. In contrast, colorimetric sensors provide simple visual readout and easier integration into flexible intelligent packaging, but their response is often semi-quantitative and can be affected by pigment stability and storage conditions.

Multifunctional platforms combining temperature, humidity, gas sensing, wireless communication, and ML-assisted data analysis may provide a promising route toward more reliable and comprehensive freshness monitoring. Nevertheless, significant challenges remain regarding long-term stability, sensor drift, fouling, environmental interference, reproducibility, and validation across different food matrices. Future research should therefore prioritize testing under realistic storage and cold-chain conditions, scalable and cost-effective fabrication, standardized performance evaluation, and appropriate safety and regulatory assessment. Addressing these aspects will be essential for translating graphene-based sensing technologies from laboratory-scale demonstrations into robust and commercially viable food-monitoring systems.

## Figures and Tables

**Table 1:** Concise qualitative overview of graphene-based sensing strategies for food freshness monitoring.

Ref.	Sensor Platform/Graphene Material	Target/Freshness Marker	Food System	Key Feature	Main Limitation
[23]	Graphene-coated Cu electrodes; dielectric spectroscopy + XGBoost	Freshness-related dielectric changes	Yak meat	Non-destructive; reagent-free; real-time	Single food matrix; external validation needed
[26]	GFET with TiO_2_ interface; FTIR/UHRMS validation	Fresh vs expired; spoilage metabolites	Agaricus bisporus	Electrical GFET readout; orthogonal validation	Single mushroom species; commercial validation lacking
[28]	PVA-PEG/rGO chemiresistive e-nose	TVB-N, NH_3_, DMA, TMA	Seafood/fish	Chemiresistive readout	RH interference; sensitivity drift
[31]	N-GQD/HNT/PVA fluorescent film	NH_3_, TVB-N	Bighead carp	Fluorescence readout; pH-insensitive design	Incomplete NH_3_ reversibility
[32]	LIG/Cu visible-light spectral sensor + SVM	Spectral signatures of chilling injury	Mango; cold chain	Integrated visible spectroscopy + ML	Ambient-light sensitivity; maintenance and scale-up cost
[34]	GDH/TiO_2_ nanofiber/rGO PEC biosensor	Glycerol/lipase (rancidity marker)	Rice bran	PEC enzymatic readout; interferent testing	Limited real-matrix and long-term validation
[36]	GO-containing QCM array + chemometrics	TMA, DMA, HCHO, NH_3_	Salmon; 4 °C	QCM array; chemometric classification	Single food model
[40]	CMC/anthocyanin/GNP/chitosan film	pH, TVB-N	Shrimp	Freshness indication + preservation function	Excess GNP reduces mechanical strength
[41]	Fish-scale gelatin/GO/anthocyanin film	pH-related color change	Wallago attu	Biodegradable, waste-derived composite	Migration and scale-up not evaluated
[42]	Basil-seed-gum/GO/Malva-anthocyanin film	pH, TVB-N	Salmon fillet	Visible halochromic readout	Pigment stability; limited moisture barrier
[43]	Soybean-polysaccharide/GO/grape-skin-anthocyanin film	pH, TVB-N	Salmon	Waste-derived dye; halochromic readout	No cross-matrix validation
[44]	Locust-bean-gum/GO/Viola-anthocyanin film	pH	Lamb meat	Biopolymer-based pH indicator	No antibacterial effect at tested formulation
[45]	CMC/GO/anthocyanin film	pH-related color change	Pork	Visual pH indicator	No cross-matrix validation
[46]	Acrylic-acid/bagasse-cellulose/GO hydrogel + citral	RH/water absorption	Mango	Humidity sensing + citral release	Fruit-to-fruit variability; real-storage validation needed
[47]	Printed gelatin/graphene humidity sensor	RH changes	Chicken and tuna packages	Flexible printed RH readout	High-RH drift not evaluated
[49]	LIG temperature sensor + heater	Temperature/frost protection	Orange and pear	Temperature sensing + active heating	External power/display; fruit-specific patch size
[51]	LIG paper gas/temperature sensor + wireless readout	TMA, temperature	Pork and milk	Package-integrated wireless dual sensing	Limited validation in real packages
[53]	PVA/anthocyanin/GNP dual-mode NFC sensor	NH_3_	Shrimp	Battery-free visual + resistive readout	Single food model; limited storage validation

**Table 2:** Quantitative key performance indicators (KPI) for the subset of studies reporting numerical data.

Ref.	Range/LOD	Sensitivity/Selectivity	Response/Recovery	Calibration/Reproducibility	Stability/Robustness
[28]	Range: 20–1000 ppm; LOD: NR	TVB-N response: 321/640/3195 at 100/200/1000 ppm; selectivity ratio: ≈1	t_resp: 440->310 s (20->1000 ppm); t_rec: 30–70 s	R^2^ = 0.99 (TVB-N, NH_3_, DMA, TMA)	T: −10 to 35 °C; sensitivity loss at 120 d: 11.2% (TVB-N), 10.4% (NH_3_), 8.6% (DMA), 9.8% (TMA); RH limit: <50%
[31]	NH_3_ range: 20–500 ppm; NH_3_: LOD: 0.63 ppm; TVB-N LOD: 9.20 mg/100 g (25 °C), 8.89 mg/100 g (0 °C)	Interferents tested: CO_2_, NO, NO_2_, H_2_S, ethanol, acetic acid; pH effect: negligible	t_exp: 3 min; t_rec: NR; reversibility: incomplete	R^2^ = 0.98	Fluorescence variation at 7 d: 7%
[34]	Lipase range: 0.02–90 U/mL; Lipase LOD: 0.043 U/mL; glycerol: range 0.025–7 mmol/L; glycerol LOD: 0.017 mmol/L	Interferents: alpha-/beta-amylase, protease, phytase; selectivity coefficient: NR	t_resp/t_rec: NR	R^2^: 0.99576 (lipase), 0.99835 (glycerol), 0.99836 (NADH); RSD: 0.99%	Signal retention: >90% (10 d); 81.37% (20 d)
[36]	Range/LOD (μmol/L): TMA 3–36/0.37; DMA 4–48/2.02; HCHO 7–84/2.10; NH_3_ 6–72/1.56	Selectivity coefficient: NR	t_rec (s): TMA 120; DMA 90; HCHO 100; NH_3_ 90; t_resp: NR	R^2^: R^2^: TMA 0.94; DMA 0.96; HCHO 0.99; NH_3_ 0.97 QSVM accuracy: 100%; r (TVB-N): 0.9782 n = 3	long-term drift: NR
[40]	pH: 2–12; LOD: NR	Sensitivity/selectivity: NR	t_visible: 2–6 h (25 +/− 2 °C); t_rec: NR	R^2^ (color difference-TVB-N): 0.98314; R^2^ (color difference-TVC): 0.98891; n = 3	Application test: 15 d at 4 °C; antimicrobial efficiency: >98%
[46]	RH: 11.30–97.60%; LOD: NR	Maximum response: 20,443% (1 kHz)	t_resp: ≈177.4 s; t_rec: ≈150.6 s	Humidity cycles: n = 6; calibration: NR	Test duration: 20 d; response drift: not significant
[47]	RH: 10–90%; LOD: NR	Sensitivity: 0.58% RH^−1^ at 10–60% RH; 2.5% RH^−1^ at 60–90% RH. selectivity NR	t_resp: 75–80 s; t_rec: 119–195 s	Inter-sensor agreement: +/−5%; n = 3	Temperature stability: ≤100 °C; bending: several hundred cycles; resistance change: negligible
[51]	TMA range: 5–40 ppm; TMA LOD: 5 ppm	Gas/temperature discrimination: signal sign and magnitude; selectivity: NR	t_resp: 51.5 s; t_rec: 65.9 s	Repeatability: n = 4; 40-ppm test duration: 7 d	Bending durability: n = 1000 cycles; encapsulation: none

NR, not reported; LOD, limit of detection; RSD, relative standard deviation; t_resp and t_rec, response and recovery times, respectively, n, number of repeated measurements or experimental replicates. Values are reported as provided in the original studies and are not directly comparable across different sensing modalities.

## Data Availability

The data are contained inside the manuscript.

## Abbreviations

The following abbreviations are used in this manuscript:

CMC	Carboxymethyl cellulose
DMA	Dimethylamine
EFSA	European Food Safety Authority
FET	Field-effect transistor
FTIR	Fourier-transform infrared spectroscopy
GDH	Glycerol dehydrogenase
GERS	Graphene-enhanced Raman spectroscopy
GFET	Graphene field-effect transistor
GNPs	Graphene nanoplatelets
GO	Graphene oxide
GQDs	Graphene quantum dots
KPI	Key performance indicators
LDA	Linear discriminant analysis
LIG	Laser-induced graphene
LIPS	Laser-induced paper sensor
LOD	Limit of detection
LSTM	Long Short-Term Memory
ML	Machine learning
MOFs	Metal–organic frameworks
NADH	Nicotinamide adenine dinucleotide, reduced form
NFC	Near-field communication
N-GQDs	Nitrogen-doped graphene quantum dots
PCA	Principal component analysis
PEC	Photoelectrochemical
PEG	Polyethylene glycol
PLA	Polylactic acid
PVA	Polyvinyl alcohol
QCM	Quartz crystal microbalance
QSVM	Quadratic support vector machine
rGO	Reduced graphene oxide
RH	Relative humidity
RSD	Relative standard deviation
SAW	Surface acoustic wave
SVM	Support vector machine
TMA	Trimethylamine
TVB-N	Total volatile basic nitrogen
UHRMS	Ultra-high-resolution mass spectrometry
UV	Ultraviolet
VOC/VOCs	Volatile organic compound(s)
XGBoost	Extreme Gradient Boosting
